# Estimating the effectiveness and cost-effectiveness of establishing additional endovascular Thrombectomy stroke Centres in England: a discrete event simulation

**DOI:** 10.1186/s12913-019-4678-9

**Published:** 2019-11-08

**Authors:** Peter McMeekin, Darren Flynn, Mike Allen, Diarmuid Coughlan, Gary A. Ford, Hannah Lumley, Joyce S. Balami, Martin A. James, Ken Stein, David Burgess, Phil White

**Affiliations:** 1 School of Health, Community and Education Studies, Northumbria University, Newcastle upon Tyne, UK; 2 School of Health and Social Care, Teesside University, Tees Valley, UK; 3 National Institute for Health Research (NIHR) Collaboration for Leadership in Applied Health Research and Care (CLAHRC) South West Peninsula, Bristol, UK; 4 Institute of Health and Society, Newcastle University, Newcastle Upon Tyne, UK; 5 Oxford University Hospitals NHS Trust, Oxford, UK; 6 Oxford University, Oxford, UK; 7 Institute of Neuroscience, Newcastle University, 3-4 Claremont Terrace, Newcastle upon Tyne, UK; 8 Royal Devon and Exeter NHS Foundation Trust, Exeter, UK; 9 Clinical Research Network North East and North Cumbria, North East and North Cumbria Stroke Patient & Carer Panel, Newcastle upon Tyne, UK; 10 North East and North Cumbria Stroke Patient & Carer Panel, Newcastle upon Tyne, UK

**Keywords:** Thrombectomy, Acute stroke, Predictive models, Health economics

## Abstract

**Background:**

We have previously modelled that the optimal number of comprehensive stroke centres (CSC) providing endovascular thrombectomy (EVT) in England would be 30 (net 6 new centres). We now estimate the relative effectiveness and cost-effectiveness of increasing the number of centres from 24 to 30.

**Methods:**

We constructed a discrete event simulation (DES) to estimate the effectiveness and lifetime cost-effectiveness (from a payer perspective) using 1 year’s incidence of stroke in England. 2000 iterations of the simulation were performed comparing baseline 24 centres to 30.

**Results:**

Of 80,800 patients admitted to hospital with acute stroke/year, 21,740 would be affected by the service reconfiguration. The median time to treatment for eligible early presenters (< 270 min since onset) would reduce from 195 (IQR 155–249) to 165 (IQR 105–224) minutes. Our model predicts reconfiguration would mean an additional 33 independent patients (modified Rankin scale [mRS] 0–1) and 30 fewer dependent/dead patients (mRS 3–6) per year. The net addition of 6 centres generates 190 QALYs (95%CI − 6 to 399) and results in net savings to the healthcare system of £1,864,000/year (95% CI -1,204,000 to £5,017,000). The estimated budget impact was a saving of £980,000 in year 1 and £7.07 million in years 2 to 5.

**Conclusion:**

Changes in acute stroke service configuration will produce clinical and cost benefits when the time taken for patients to receive treatment is reduced. Benefits are highly likely to be cost saving over 5 years before any capital investment above £8 million is required.

## Background

Endovascular thrombectomy (EVT) in the treatment of acute ischaemic stroke resulting from large artery occlusion (LAO) significantly improves independent (good) outcomes with a modified Rankin Scale (mRS) score of 0–2 - from 25 to 50% [1]. It has been shown to be cost-effective, but requires specialist facilities, expertise and training. Consequently, EVT is available only in comprehensive stroke centres (CSCs) and is not currently equally accessible to the entire English population. Structural inequalities describe situations where patients with similar capacities to benefit from treatment do not, because of the way services are organised. In the case of EVT, one goal, amongst others, of health care providers such as NHS Commissioners is to reduce structural inequalities by mitigating the effects of geography on outcomes, in particular due to differences in time to initiation of treatment.

Currently EVT rates (5.5 per 1000 ischaemic strokes) place the United Kingdom on a par with countries in Eastern Europe and the Balkans and behind Western Europe, where EVT rates can exceed 50 per 1000 ischaemic strokes [2]. In 2018–19, 1200 EVT procedures were recorded in England, Wales and Northern Ireland in the national stroke audit (SSNAP) (approximately 1.4% of all ischaemic strokes) [3]. To address this shortfall in provision, the NHS England Long Term Plan describes the objective of a 10-fold increase in this figure by 2022 [4]. To achieve this both the number of comprehensive stroke centres will need to increase as well as then numbers of EVTs carried out at these centres. A more detailed analysis of the path to achieving this figure is provided in “Thrombectomy: An Implementation Guide for Commissioners & Healthcare Providers” [5].

In a previous modelling study [6] we identified, by means of genetic algorithms, an optimal configuration for a ‘Drip and Ship’ paradigm across England, aiming to initiate definitive treatment (intravenous thrombolysis [IVT] or EVT) as soon as possible. In England, the ‘Drip and Ship’ paradigm denotes a treatment regimen in patients in whom IVT is initiated at the nearest primary stroke centre (PSC) and transferred to a CSC as soon as possible. We assumed that 50 such PSCs exist in England reflecting current policy reconfiguration intentions. The algorithm used in our previous paper identified the need for seven new comprehensive stroke centres and the downgrading of one by means of a genetic algorithm. The algorithm identified which of the existing primary stroke centres should be upgraded by looking at the geography of their constituency of patients. To prevent the algorithm identifying an optimal provision that involved upgrading every primary stroke centre to a comprehensive stroke centre, it was subject to a maximum and minimum number of stroke patients (hence EVTs) that any potential comprehensive stroke centre could undertake. These were based on national guidelines that recommend the minimum number of admissions to a primary or comprehensive stroke centre is 600 patients per year [7] and that travel to first point of stroke care should be ideally 30 min or less, and no more than 60 minutes [8]. Additionally; to provide a robust 24/7 EVT service realistically requires at least five operators, and all five could not hope to meet minimum activity levels to maintain competence if EVT volume was less than 150 procedures. There is no guideline on the maximum size of a primary or comprehensive stroke centre, but NHS England guidance recommends a maximum of 1500 admissions for a single team, and the largest centre currently in the England has about 2000 admissions [9].

We concluded that, subject to the model constraints, one optimal solution would be to increase the number of CSCs providing EVT in England from 24 to about 30, by closing one clinically unsustainable centre in London and opening seven new centres across England. With 30 CSCs, structural inequality was substantially reduced; 52% of the population in England would be within 30 min travel of a centre compared with 43% (82% versus 71% within 45 min, and 93% versus 86% within 60 min), reducing the impact of geography on outcomes. For the remainder, these constitute sparsely populated areas, distant from CSCs where helicopter transfer may provide a better solution to reducing inequality [10].

## Methods

### Aim

By means of an economic model, we aim to provide additional information to support the implementation of recommendations for EVT service expansion [11]. Specifically, to complement previous estimates of sustainability and increased equity, we sought to estimate the health economic impact (clinical effectiveness and cost-effectiveness) of increasing the number of CSCs from 24 to 30. Clinical effectiveness was estimated in terms of the changes in mRS in treated patients and cost-effectiveness in terms of cost per quality adjusted life- year (QALY) from a payer perspective.

### Modelling approach

Informed by development work undertaken from our previous modelling [6, 12], we developed a discrete event simulation (DES) model to estimate the relative effects and cost-effectiveness that accrue from increasing the number of CSCs centres in England. Discrete Event Simulations are one of the most common modelling techniques and, its use is increasing in Health Technology Assessment (HTA) [13–16]. We chose DES as the modelling technique in preference to Markov models [17]. The latter typically models relatively crude changes in mRS categories (0–2, 3–5 and 6) and is computationally burdensome; whereas a DES enables modelling across each of the seven mRS states relatively effectively. A DES creates one estimate of the time an event occurs rather than a set of probabilities of an event occurring over time. Unlike a Markov model, probabilities of events are not estimated at regular intervals; in a DES events occur according to their probabilities of occurrence over time. The DES was iterated 2000 times for each person in the population that would be affected by the change in service configuration. The mean outcomes were aggregated to estimate the marginal effects, before and after proposed service change, at the population level, as well as to quantify uncertainty around these estimates.

### Model structure and assumptions

**Fig. 1:**
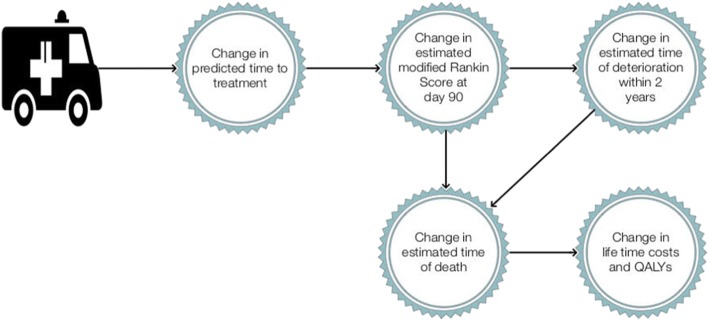
Model Overview

The DES starts with a LAO stroke patient whose 90-day outcomes are solely dependent on time to treatment. It then includes two post-90 day events; increase in dependence as measured by deterioration in mRS by 2 intervals and mortality (mRS =6). A depiction of a LAO stroke patient in the DES is shown in Fig. 1. For those patients alive at 90-days; an event occurs at a random time in the future; where time of event is a function of a patient’s 90-day mRS status. If that event is further deterioration in mRS, then death occurs at a subsequent time in the future based on age and subsequent mRS status which increases mortality risk. For the purposes of this DES model, deterioration could only occur in years 1 to 5 and resulted in an increase in mRS.

The DES repeatedly modelled each of the affected stroke patients’ journeys and the mean costs and effects were used as estimates of lifetime cost-effectiveness. Each repeated simulation drew a random set of parameters to reflect the uncertainty around eligibility, journey times, costs and outcomes. Each simulation equates to a single point on a cost-effectiveness plane for the entire cohort of patients influenced by the service change. In this DES model, EVT ineligible patients would be unaffected by the change in EVT provision and were excluded from the simulation to reduce computational burden. The benefit of shorter times to EVT provision accrues in the model only for early presenters (presenting ≤270 min since onset) with National Institutes for Health Stroke Severity Scale score (NIHSS) ≥6, through shorter times to treatment; this and other fixed assumptions of the model are listed in Table 1.

**Table 1:** Fixed Modelling Assumptions

Data	Assumption
Primary Stoke Centres (PSC) in addition to CSCs	FIXED at 50 in both scenarios
Stroke incidence in England	FIXED at 80,800 For compatibility with model predicting optimal number of CSCs centres [6, 19]
Early presenters presentation times	FIXED to the distribution of those moderate and severe (NIHSS ≥6) confirmed LAO strokes presenting to a large Hospital in North East England^a^.
Stroke patient characteristics	FIXED to those of the HERMES Meta-Analysis cohort [18]
Distances	FIXED road distances calculated by Geographic Information System (GIS) from geographic population weighted centroid of Local Super Output Area of patients home or between locations of PSU and CSC [6]
Time dependent outcomes of EVT	FIXED to those of the HERMES Meta-Analysis cohort [18]
Early presenters	FIXED at those initially presenting to either a PSU or an EVT CSC within 270 min of stroke onset [19].
Stroke mimics	No mimics are included in the simulation
Death	Is determined only by mRS at 90 days or following subsequent deterioration
Deterioration	Only occurs within 5 years and is determined only by mRS at 90 days and is FIXED at a two point increase in mRS to a maximum of 5.
Door in Door Out	FIXED at 60 min, determined by optimisation [6]
Door to EVT time	FIXED at 90 min, determined by optimisation [6]
Maximum Lifetime	FIXED at 100 years

^a^Northumbria Healthcare median onset to door time for all LAO with NIHSS > = 6 is 180 min, IQR 80–690 min

The simulation was implemented in R as 2-dimensional matrix: The first dimension representing patients, where each EVT eligible patient was included 2000 times representing the number of simulation runs. The second dimension represented the patients attributes including invariant data items such as age, sex and variant items such as time to treatment, the main predictor of clinical outcome, under each scenario and times and types of events under each scenario. The use of parametric survival functions mean the DES runtime was effectively the life time of each patient, although this was truncated when a patient reached 100 years. For memory efficiency, the data.table package was used. The DES effectively consisted of the creation of this matrix, its manipulation (by a set of R functions) and its processing to report outcomes. No other libraries or packages were used. The matrix construct was chosen as it allowed the use of vector operations.

The model’s empirical internal validity was assessed deterministically by testing both the individual components of the model and the processing of the matrix to estimate the marginal cost and QALY implications: Modelled outcomes (for 90 day modified Rankin, survival, deterioration and consequent costs and QALYs) were compared to a values computed independently of the model.

In keeping with our previous study that identified the potential location of the additional centres, no changes in IVT pathways were assumed to occur. However, we assumed a reduction of 30 min between IVT and EVT procedure when patients did not require transfer from a primary stroke centre (PSC) to a CSC.

### Data sources

We utilised data from Northumbria Healthcare NHS Foundation Trust (NHFT) with moderate to severe LAO stroke to reflect variability in early presenters’ onset to door time, whilst maintaining the differential in time to treatment due to differing secondary transfer times in each scenario. NHFT serves a mixed population - the majority live in urban or semi-urban locations near to stroke units, and a minority live in more distant rural areas. We obtained anonymised data about 600 consecutive patients presenting with NIHSS scores of ≥6 with confirmed LAOs. Of these, 440 patients presented early (≤270 min) with a median presentation of 85 min (IQR 60–133) [19]. During each of the 2000 simulations, we sampled with replacement from this distribution to estimate initial presentation time of an early presenting patient.

For patients treated with EVT, short-term outcomes were defined by mRS using results of the HERMES meta-analysis [18]. Early or late presentation was defined at initial presentation, which would not change by upgrading a PSC to a CSC centre or vice versa. Outcomes for late presenters (> 270 min from onset) were not estimated in our model as outcomes were assumed to be relatively time invariant; no published information yet exists about the association between time to treatment and outcome in “all comer” late presenter populations: The DAWN trial [20] considered outcomes in patients presenting between 6 and 24 h and estimated absolute treatment effect in this period based on clinical imaging mismatch and independently of time to treatment. This small group (DAWN &/or DEFUSE-3 eligible) is therefore excluded in our model of marginal effects [18, 21].

Simulation parameters are shown in Table 2. Long-term outcomes were those reported in the Oxford Vascular Study (OXVASC) [22] study which estimated survival from 90 day mRS for patients experiencing a major stroke [23]. We estimated death by combining the results of OXVASC study (for mortality 90 days to five years after stroke) with mortality data from national life tables [24] and data from the Lothian stroke register [25] about the increased mortality associated with stroke survivors depending on their 90 day mRS, using a knotted spline technique to extrapolate survival [26]. This generates a different set of extrapolated parametric survival curves for each iteration of the simulation from which the time to death could be calculated from the uniform randomly drawn probability of death [27]. To allow for improvements in survival since the Lothian stroke register data was collected, we applied a reduction in mortality of 25% from year 6 onwards. This was obtained from a longitudinal study that reported composite mortality rates after stroke falling by 20% from 9.3 to 7.4% [28]. The mean median and inter-quartile range survival for each mRS across all simulation runs, for a 70 year old stroke patient, based on this is shown in Table 2. Our model also allowed for deterioration within the first five years. We included data from OXVASC study about increases in dependency, as measured by mRS by calculating an annualised probability of an increase of at least two points if the 90 day mRS was 3 or less, and 1 if 90 day mRS was 4 or more. Combining this with survival data resulted in a combined probability of death or deterioration, with a further random variable used to determine whether deterioration or death were the first event experienced during each simulation; Each probability was rescaled to sum to one (i.e. a 20% chance of death at the time the event occurred and a 5% chance of deterioration were rescaled to 0.8 and 0.2) and the event decided by a random draw between 0 and 1.

**Table 2:** Simulation Parameters

Parameter	Mean and Uncertainty; distribution and parameters	Source
Cost of EVT	£9116 (£2519); gamma (554.86, 16.42)	Balami [20]
Cost of Category A ambulance per minute	£6.86	PSSRU [18]
Survival (years) following stroke at 70		
mRS 0, median (IQR)	8.4 (4.7,14.1)	Estimated from results of
mRS 1, median (IQR)	7.9 (4.3, 13.2)	DES^a^
mRS 2, median (IQR)	7.2 (3.8, 12.3)	
mRS 3, median (IQR)	3.7 (1.4, 7.0)	
mRS 4, median (IQR)	2.7 (0.92, 5.8)	
mRS 5, median (IQR)	1.3 (0.42, 3.6)	
mRS 6, median (IQR)	NA	
Utilities		
mRS 0	0.95, 0.08; beta (48.4, 2.55)	MR CLEAN [22]
mRS 1	0.93, 0.13; beta (128.04, 9.64)	
mRS 2	0.83, 0.21; beta (222.24, 45.52)	
mRS 3	0.62, 0.27; beta (173.70, 106.46)	
mRS 4	0.42; 0.28; beta (173.15, 239.11)	
mRS 5	0.11; 0.28; beta (6.07, 49.12)	
mRS 6	0	
Cost Year 1		
mRS 0	£6620	Dewilde et al. [17]
mRS 1	£11,196	
mRS 2	£18,929	
mRS 3	£35,771	
mRS 4	£60,118	
mRS 5	£60,458	
mRS 6	£0	
Yearly Cost Thereafter		
mRS 0	£2122	Dewilde et al. [17]
mRS 1	£2836	
mRS 2	£4722	
mRS 3	£12,291	
mRS 4	£30,750	
mRS 5	£28,853	
mRS 6	£0	
Proportion all strokes presenting early with LAO & NIHSS ≥6	10.6% (SD 0.1%)	McMeekin et al. [23]
Monthly probability of deterioration^b^ (increased mRS) before year 6		Rothwell et al. [10]
0	0.006	
1	0.004	
2	0.002	
3	0.001	
4,5	As mortality	

^a^Estimated from modelled mortality based on OXVASC, UK lifetables, Lothian Stroke Register

^b^Two or more point increase in mRS

Costs associated with each level on the mRS in the first year after stroke and subsequent years came from a longitudinal study of 569 stroke patients in Belgium [29], converted to British Pounds (sterling) using the Organisation for Economic Co-operation and Development (OECD)purchasing parity index [30] and inflated to 2017 prices using the Hospital and Community Health Services (HCHS) index [31]. The cost of EVT were derived from a UK micro costing study [32], ambulance costs from the Personal Social Services Research Unit (PSSRU). The uncertainty around cost estimates is captured by using a gamma distribution to reflect the right skewed nature of the data. Where no standard deviations for costs were available the method of Briggs et al. [33] was used, whereby the standard error was assumed to be the same value of the mean. Utility scores which reflect quality of life were modelled using a beta distribution to reflect their bounded nature between zero and one and were those reported in the MR CLEAN study [34]. Random draws of cost and utilities associated with mRS were generated using copulas to maintain the correlation of the parameter. A copula is a multivariate probability distribution where the marginal probability distribution of each variable is uniform and typically used to describe the dependence between random variables. For example, a simulation run never occurs where the utility associated with mRS 3 is greater than mRS 2.

### Reporting clinical effectiveness and cost-effectiveness

In reporting clinical effectiveness, the DES model estimates the mean number of patients whose mRS scores would change as a result of service reconfiguration. Similarly, in reporting cost-effectiveness, the marginal cost and the marginal QALYs for each simulation run are used to calculate the incremental cost-effectiveness ratio (ICER). The mean costs and mean QALYs represent the outcomes across the patients that are affected by the EVT service reconfiguration.

### Uncertainty and sensitivity analyses

For visualising the uncertainty around clinical effectiveness, a plot shows the estimated number of patients with changes in mRS scores if EVT service reconfiguration is adopted. The cost-effectiveness of each simulated outcome across the cohort are shown on an incremental cost-effectiveness plane. Uncertainty around cost-effectiveness (cost per QALY) is explored by means of a cost-effectiveness acceptability curve (CEAC) which shows the probability that the proposed EVT service reconfiguration is cost-effective at increasing willingness-to-pay thresholds. QALYs that result from the proposed reconfiguration are valued at the threshold and added to any cost savings to estimate a net financial benefit. Furthermore, the proportion of simulations of the service reconfiguration whose net benefit fall below the threshold represents the probability that is cost-effective. If a simulation is cost-saving and results in a QALY loss, those QALYs are also valued at the threshold and included in the net benefit calculation.

The Willingness to Pay (WTP) for a QALY is derived from the notion of acceptable thresholds of cost-effectiveness. It is a construct that monetises on health gains which are added to any financial savings or consequences of healthcare intervention. The greater the willingness to pay (WTP) for a QALY, the more likely an intervention that generates health gains is to be deemed cost effective. There is no explicit threshold, but in England the WTP for a QALY is estimated to be between £20,000 and £30,000 [35]. These amounts have been inferred from decisions taken by the National Institute for Clinical Effectiveness.

We also undertook three one-way sensitivity analyses. Firstly, we replaced cost per mile with the current estimated average Ambulance mission Tariff to £234 [30]. Secondly, we varied by ±1% the proportion of early presenting patients with moderate-to-severe LAO strokes and finally we varied the age of patients by ±5 years. The average age at first stroke, for all sub-types, in the UK was reported in 2008 as 77 years in women and 71 years in men [ 36] and therefore older than the patients in the HERMES meta-analysis that formed our base case. Our model assumes that the patients included in our simulation had the same properties as those included in the HERMES [19] analysis of time to treatment: 66 years we varied the age of patients within our simulation, the only effect of this was on post 90 day survival and deterioration. We implicitly assumed that EVT outcomes for eligible patients were invariant with age across the range of our sensitivity analysis.

The DES was created in R and the flexsurv library was used to fit appropriate parametric functions to survival data. The copula library was used to generate random multivariate distributions using copulas and parametric margins.

## Results

The results of the simulation are summarised in Table 3. We estimate that 21,740 stroke patients would be affected by the change in service configuration from 24 to 30 CSCs with a fixed number of 50 PSCs in England. Of these the mean number of patients treated with EVT would be 2540 (SD 7); the median time to treatment for (EVT eligible) early presenters would reduce from 195 (IQR 155–249) to 165 (IQR 105–224) minutes. 2316 (SD 4.5) patients would benefit from shorter secondary transfer times but 222 (SD 2.0) patients would additionally face a secondary transfer (due to the service reconfiguration from 24 to 30 by creating 7 new centres and closing an existing one).

**Table 3:** Simulation Results

Outcome	Estimate
Mean time to treatment reduction (SD)	42 min (SD 63)
Changes in population mRS (n) *	
0	15
1	18
2	-4
3	-10
4	−8
5	−8
6	−4
Marginal Lifetime QALY gains across English pop.	213 (95% CI 28, 447)
Marginal Lifetime costs to NHS England	-£2,870,000 (95% CI -£7,946,000 to £2,051,000)
Net Benefit at £20,000 per QALY^b^	£7,123,000 (95% CI £1,039,000 to £13,666,000)
Net Benefit at £25,000 per QALY^b^	£8,187,000 (95% CI £1,609,000 to £ 15,684,000)
Net Benefit at £30,000 per QALY^b^	£ 9,250,000 (95% CI £1,983,000 to £ 17,532,000)
Budget Impact Analysis	
Year 1	-£981,000(95% CI -£2,067,000 to £218,000)
Years 2 to 5 (discounted)	- £1,186,000 (95% CI -£3,587,000 to £1,187,000)
Sensitivity Analyses expressed as change in Net Benefit at £25,000 willingness to pay for QALY	
Use of Ambulance Tariff	£20,000
1% increase in LAO incidence	£93,000
1% decrease in LAO incidence	-£72,000
Mean age at stroke −5 years (65)	£1,023,000 22 additional QALYs, additional savings of £473,000
Mean age at stroke + 5 years (75)	-£934,000 27 fewer QALYs, reduction in savings of £259,000

^a^Rounded to nearest number of patients

^b^Net benefit is calculated by deducting the ‘value’ of QALYs generated from increased costs

**Fig. 2:**
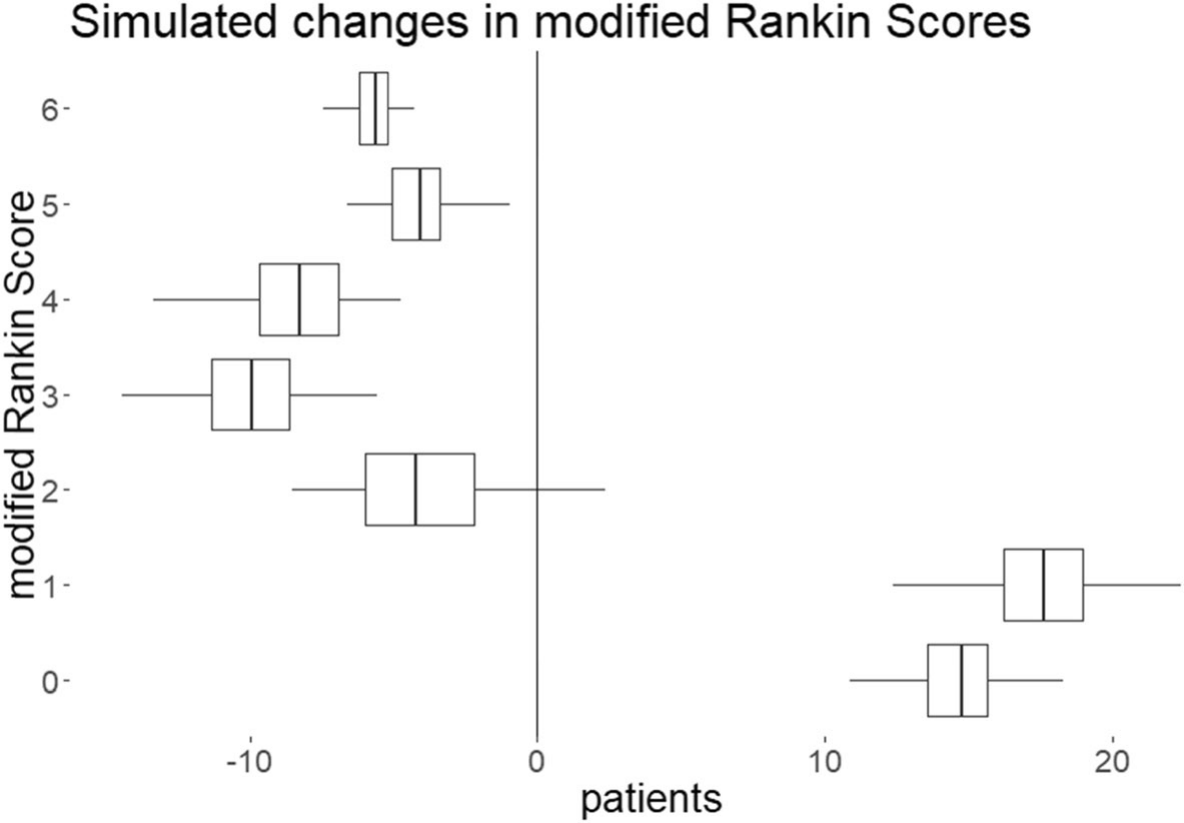
Modelled Changes in Outcomes

**Fig. 3:**
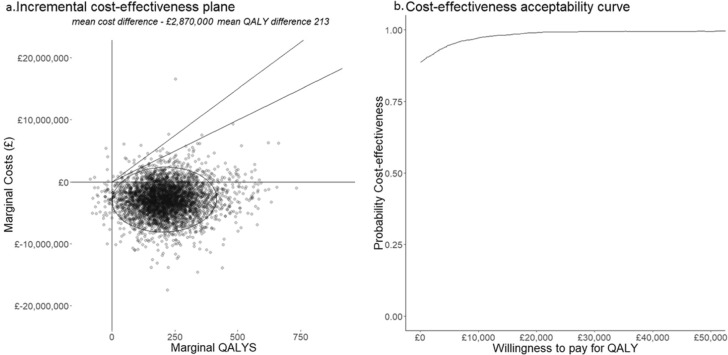
Incremental Cost-Effectiveness Plane and Cost Effectiveness Acceptability Curve

The effectiveness and cost-effectiveness results are presented graphically in Figs. 2 and 3. Across the 2000 simulations, reconfiguration from 24 to 30 CSC results in 32 individuals estimated to benefit from reduced dependency or death (Table 3 and Fig. 2). 14 of these achieve a mRS of zero. The cost-effectiveness plane (Fig. 3a), where each of the 2000 points represents one set of aggregate outcomes for the affected population, shows that QALY gains are not always associated with cost savings, that the savings made from reduced dependency might not always offset the costs of ongoing care created by avoidance of death. The ellipse represents the area that 95% of these scenarios occupy and the majority represent the doubly beneficial situation where QALYs are generated and costs are saved. The two rays from the origin of the cost-effectiveness plane represent £20,000 and £30,000 WTP thresholds. Points under these lines would be deemed cost-effective at the relevant WTP threshold. The lower line represents £20,000 per QALY and the upper £30,000; at each of these, the reconfiguration would be considered highly likely to be cost-effective.

Figure 3b shows the CEAC at willingness to pay thresholds for QALYs from £0 to £50,000. At £0 this is 89%, reflecting the number of simulations resulting in costs in the lower-left, cost-saving and QALY positive, quadrant of the cost-effectiveness plane, Fig. 3a. Uncertainty about cost-effectiveness is not reached at any threshold because of simulations that result in estimates in the south-west quadrant. As willingness to pay for a QALY increases, the financial compensation required for a lost QALY rises. As willingness to pay increases, willingness to accept increases, more simulations in the upper-right quadrant become cost effective and fewer simulations in the lower-left quadrant remain cost effective.

The results of the one-way sensitivity analyses (Table 3) show that using a common tariff for secondary transfer, did not change QALY outcomes and decreased net benefit by £20,400, reflecting the lower average costs and transfer distances associated with a per mile cost compared with the common ambulance tariff. Increasing the proportion of patients eligible for EVT presenting early from 10.6 to 11.6% increased net benefit to £92,700. This resulted from an additional 3 QALYs and a further saving of healthcare costs of £17,000. Decreasing eligibility to 9.6% reduced QALY gains by 2 and increasing healthcare costs by £21,600 resulting in a £71,600 loss in net benefit. Reducing the mean age of patients from 70 to 65 increases QALY gains by 22 and increases health and social care savings by £472,700; resulting in an increase in net benefit of £1,022,700. Conversely, increasing the age of patients to 75 results in 27 fewer QALYs and reduces savings by £259,500. This equates to reduction in net benefit of £934,500.

## Discussion

Increasing the number of CSC providing EVT in England from 24 to 30 would deliver health gains within current thresholds of cost-effectiveness with a very high probability of being cost-effective. The magnitude of overall gains in health and in cost savings are driven by significant changes in outcomes in a small number of patients. Our model shows that, as expected, a reduction in time to EVT is characterised by a shift towards better outcomes. Our results confirm that net benefit is significantly influenced by the costs of future healthcare, and in turn this is influenced by the longer term mortality of stroke patients. Our model was only sensitive to patient age beyond 90 days, 90 day outcomes were those of HERMES meta-analysis. If increasing age was associated with poorer outcomes, the modelled decreased decrease in benefit may be even greater.

The modelled effectives in terms of improved mRS scores and cost-effectiveness in terms of cost per QALY and net benefit should be considered along with information about reductions in structural-inequity and budget impact that the proposed change in service configuration delivers. Our model shows that in each year, a saving of £1 million pounds will be made because of improved outcomes in patients treated that year. In years 2 to 5 these improved outcomes will result in a further £1.2 million saving equating approximately to a £8 m return on investment over five years. Currently commissioners must also consider reductions in structural inequality alongside cost-effectiveness without information about how society would value any reduction. In a parallel ongoing study we intend to elicit societal preferences in order to ‘value’ these reductions, and thereby include them in a subsequent net benefit calculation using our DES model.

### Strengths and limitations

Our model is the first DES based economic evaluation to use individual mRS scores as outcomes rather than crude changes in mRS categories. Our model is therefore more in line with outcomes used in randomised control trials and more relevant to routine stroke care. Our analysis took a payer perspective and ignored implications to the wider economy. In an employed population an outcome of mRS 0 suggests that a patient would be able to return to work, and as such it would be feasible to include wages not forgone if the analysis were from a societal perspective. The use of population mortality data in combination with mRS related mortality allows the model to predict longer-term outcomes.

Our analysis used as an exemplar, one potential optimised configuration of 30 CSCs derived from our earlier geographical modelling work, chosen from among many possible options [6]. The new configuration involved closing down one unit, which means that some patients experienced longer times to treatment and concomitant worse outcomes. We included simulations which result in scenarios where the outcome of reconfiguration resulted in both cost savings and QALY losses as generating a net benefit. Because over each simulation run the costs and QALYs were summed to estimate the net effect and because of the 7:1 ratio of centres opening to centres closing, at the population level only a small number of scenarios resulted in cost savings and QALY losses, which were never judged cost-effective at any threshold. However, at the individual patient level within a simulation summing QALY losses and QALY gains, it is equivalent to assuming an equal value of a QALY lost as a QALY gained. If society places more value on a QALY lost than it would pay for a QALY gain, our estimates of net benefit are biased in favour of the new service configuration. In contrast, we also assumed that, in the absence of other information that the decline in treatment effect of EVT over time was linear. If the relationship between effect and time is not linear, as for thrombolysis for ischaemic stroke, then our model potentially underestimates the gains from expedited access to EVT: The benefits estimated in the HERMES meta-analysis included some patients with salvageable brain tissue identified by advanced imaging and therefore more likely to benefit. These patients tended to be among those treated later and this will contribute to an underestimate of the decline in treatment effect over time. Outcomes for LAO treated by EVT were estimated from the data from the HERMES group, and as such our estimated outcomes are those of a 70 year old patient and do not account for uncertainty over time. Changing the age of patients in our model takes no account of outcome of EVT dependent on age, and simply increases benefit due to increased life expectancy. As a consequence the uncertainty in our model is only that of financial consequences and post 90-day mortality and morbidity. Our model also ignored any benefits that might accrue to patients presenting outside the thrombolysis window. Whilst there is no published estimates of how benefit changes with time to treatment in this group, benefits may result from quicker access to treatment. Furthermore the additional thrombectomy centres may be better placed to identify eligible late presenters and provide treatment.

Although out model has seven eventual outcome states, it does not reflect changes within states and therefore underestimates treatment gains as improvements within mRS states are not accounted for in the DES. This is a feature of a DES, however the mean costs and effects should not differ from a Markov Model where individuals are apportioned across states. Our model includes no costs associated with setting up new centres. This is equivalent to assuming that investments to upgrade centres can be financed through income; however, that is the actual approach NHS England has taken to introducing EVT of LAO stroke as a routine service into the existing 24 English CSCs.

## Conclusion

Increasing the number of CSCs in England from 24 to 30 would be effective and highly likely to be both cost-effective and cost-saving. The net benefit from patients treated in one year to the NHS would be between £7 million and £9 million. In cash terms the savings are estimated to be over £2 million over five years. The proposed reorganisation would therefore yield a return on investment over five years of £8 million, notwithstanding initial capital costs to establish new centres. The magnitude of savings is dependent on the longer term survival of stroke patients and less sensitive to the numbers of eligible patients and the costs associated with ambulance transfer.

## Data Availability

All data except the summary data from Northumbria Healthcare NHS Foundation trust was taken from the literature and is referenced in the manuscript.

## Abbreviations

CEAC: cost-effectiveness acceptability curve
CSC: comprehensive stroke centres
EVT: endovascular thrombectomy
HCHS: Hospital and Community Health Services
HERMES: Highly Effective Reperfusion Using Multiple Endovascular Devices Collaboration
HTA: Health Technology Assessment
ICER: incremental cost-effectiveness ratio
LAO: Large Artery Occlusion
mRS: modified Rankin score
NHFT: Northumbria Healthcare NHS Foundation Trust
NHS: UK National Health Service
NIHR: National Institute for Health Research
OECD: Organisation for Economic Co-operation and Development
PISTE: `The Pragmatic Ischaemic Thrombectomy Evaluation trial
PSC: Primary Stroke Centre
PSSRU: Personal Social Services Research Unit
QALY: Quality Adjusted Life Year
STABILISE: Stroke: an evaluation of thrombectomy in the ageing brain
WTP: Willingness to pay
